# Testing Machine Learning Models to Predict Postoperative Ileus after Colorectal Surgery

**DOI:** 10.3390/curroncol31060262

**Published:** 2024-06-19

**Authors:** Garry Brydges, George J. Chang, Tong J. Gan, Tsuyoshi Konishi, Vijaya Gottumukkala, Abhineet Uppal

**Affiliations:** 1 Division of Anesthesiology, Critical Care & Pain Medicine, The University of Texas MD Anderson Cancer Center, Houston, TX 77030, USA; gbrydges@mdanderson.org (G.B.); tjgan@mdanderson.org (T.J.G.); 2 Department of Colon & Rectal Surgery, The University of Texas MD Anderson Cancer Center, Houston, TX 77030, USA; gchang@mdanderson.org (G.J.C.); tkonishi@mdanderson.org (T.K.); auppal1@mdanderson.org (A.U.); 3 Department of Anesthesiology & Perioperative Medicine, The University of Texas MD Anderson Cancer Center, Houston, TX 77030, USA

**Keywords:** colorectal cancer, comorbidities, postoperative ileus, machine learning, neural networks, artificial intelligence

## Abstract

**Background:** Postoperative ileus (POI) is a common complication after colorectal surgery, leading to increased hospital stay and costs. This study aimed to explore patient comorbidities that contribute to the development of POI in the colorectal surgical population and compare machine learning (ML) model accuracy to existing risk instruments. **Study Design:** In a retrospective study, data were collected on 316 adult patients who underwent colorectal surgery from January 2020 to December 2021. The study excluded patients undergoing multi-visceral resections, re-operations, or combined primary and metastatic resections. Patients lacking follow-up within 90 days after surgery were also excluded. Eight different ML models were trained and cross-validated using 29 patient comorbidities and four comorbidity risk indices (ASA Status, NSQIP, CCI, and ECI). **Results:** The study found that 6.33% of patients experienced POI. Age, BMI, gender, kidney disease, anemia, arrhythmia, rheumatoid arthritis, and NSQIP score were identified as significant predictors of POI. The ML models with the greatest accuracy were AdaBoost tuned with grid search (94.2%) and XG Boost tuned with grid search (85.2%). **Conclusions:** This study suggests that ML models can predict the risk of POI with high accuracy and may offer a new frontier in early detection and intervention for postoperative outcome optimization. ML models can greatly improve the prediction and prevention of POI in colorectal surgery patients, which can lead to improved patient outcomes and reduced healthcare costs. Further research is required to validate and assess the replicability of these results.

## 1. Introduction

Despite a few patient comorbidity risk predictive instruments used for identifying postoperative morbidity and mortality, identifying specific patient comorbidities and conditions contributing to postoperative ileus (POI) continues to challenge clinicians’ efforts to optimize patient care. Distinguishing between early bowel obstruction and paralytic POI (without obstruction) is crucial for optimal management, as their etiologies differ. Prolonged gastrointestinal paralysis exceeding expected recovery times not only delays oral intake and increases hospital stays but also incurs significant economic burdens, estimated at USD 1.5 billion annually in the United States alone [1]. Albeit, POI is not the most critical postoperative complication. Identifying and preventing life threatening complications, such as anastomotic leaks and sepsis remain a priority, but POI does impede patient recovery and contribute significantly to healthcare costs [2]. Comorbidity risk instruments that reliably predict postoperative morbidity, such as POI, in the early perioperative period have variable accuracy [1]. Many comorbidity risk instruments exist to help predict risk across the perioperative continuum, such as ASA physical status, the National Surgical Quality Improvement Program (NSQIP) index, the Charlson Comorbidity Index (CCI), and the Elixhauser Comorbidity Index (ECI). Some instruments include many comorbidity variables, but some essential variables may be absent in other instruments, contributing to inter-instrument variability, with significant differences between instruments on the same dataset as demonstrated in a comparison of NSQIP to University Health Consortium (UHC) models (13% vs. 1% risk of surgical site infection) [2,3]. 

Advanced machine learning (ML) models may mitigate the limitations of traditional risk indices by identifying and capturing non-linear relationships within large datasets. During the ML training process, less influential variables are gradually filtered out or down weighted, while non-linear relationships of more significant variables are refined and amplified [3,4]. The ML process allows for analyzing large datasets by simultaneously leveraging the power of multiple advanced statistical techniques. These ML methods are often used in clinical bioinformatics to predict postoperative outcomes from patient characteristics and laboratory values collected before surgery [4,5]. ML models offer a promising approach to overcome existing limitations in predicting POI. In contrast to traditional risk assessment tools based on predefined factors (i.e. NSQIP, CCI, ECI), ML algorithms can process and analyze extremely large amounts of patient data, identifying complex patterns and relationships influencing the risk of developing POI. Additionally, ML models can learn from large datasets and enhance their predictive accuracy over time [1,4,5,6].

Predicting POI after colorectal surgery remains a challenge. Matsui et al. identified several risk factors for POI, including right colon resection, pre-operative chemotherapy, antithrombotic drugs, and severe postoperative complications [6]. However, predictive accuracy remains limited due, in part, to inconsistent POI definitions and diagnostic methods across studies, leading to potential underdiagnosis, even for relatively common symptoms like absent defecation. Identifying modifiable and non-modifiable risk factors paves the way for improved POI prediction strategies. Pre-operative assessment, considering these factors, could help identify high-risk patients for closer monitoring and preventive measures. Vigilant postoperative surveillance for early signs of POI in high-risk patients could enable prompt intervention, shortening the duration of POI. POI is a common complication after colorectal surgery and can prolong hospital length of stay (LOS) [6]. In this study, we used several machine learning models to identify patient characteristics and comorbidities potentially associated with developing POI after colorectal surgery and compare ML model accuracy to existing predictive risk instruments [6].

## 2. Materials and Methods

### 2.1. Study Design and Participants

From an MD Anderson Cancer Center Institutional Review Board-approved (2022-0002; 26 January 2022) colorectal surgery database, adult patients who underwent colorectal surgery from January 2020 to December 2021 were included in this retrospective study. To reduce heterogeneity of the cohort, patients undergoing multi-visceral resections, re-operations, or combined primary and metastatic resections were excluded. Type of operation and approach (open or minimally invasive) were identified from operative notes. Patients lacking follow up within 90 days after surgery were excluded. After exclusions, the sample included 316 patients.

### 2.2. Data Acquisition and Variable Selection

Data were collected from a data warehouse including electronic health record (EHR) information, quality databases, and financial analytics information. Dedicated surgical subspecialty quality databases function as comprehensive repositories for collecting granular procedural data. These databases empower rigorous analysis of surgical processes within specific fields like colorectal surgery. By facilitating the identification of modifiable factors influencing patient outcomes, these quality databases enable evidence-based refinement of perioperative care standards. 

The dataset contained 29 comorbidities, which were coded as binary variables (1 = present or 0 = absent). The comorbidity risk index scores utilized include ASA physical status, NSQIP, CCI, and ECI. All patients received standard preoperative evaluation by anesthesiology and surgical services for surgical and anesthesia risk evaluation. Patients presenting with significant comorbidities were further evaluated through a perioperative evaluation and management (POEM) center. Patient comorbidities were identified from billing codes and clinical documentation at their pre-operative visit. The target outcome, POI, was categorized as present or absent during the hospital stay based on clinical documentation and ICD-10 codes (K56.0 and K56.7). Length of stay was calculated from date of surgery to date of discharge. Readmission within 30 days and reason for readmission were identified from clinical documentation and ICD-10 codes [7].

### 2.3. Statistical Analysis

This study adheres to established scientific principles within standard ML pipelines, applying synthetic minority oversampling technique (SMOTE), rigorous regularization techniques, and statistical performance evaluation for objective and reproducible results. Table 1 delineates the conventional ML methodology employed for addressing imbalanced datasets.

The categorical variables were reported as frequencies and percentages. The continuous variables were tested for normality using the Shapiro–Wilk test. For age, the *p*-value = 0.055 was a marginal departure from normality, but age was treated as normally distributed. Length of stay (LOS) (*p*-value = 0.00), BMI (*p*-value = 0.00) and NSQIP (*p*-value = 0.00) were not normally distributed for *p*-values less than 0.05. Thus, age was reported as mean and standard deviation. LOS, BMI, and NSQIP were reported as median and range. Associations between POI and the other variables were analyzed using ML algorithms to identify a potential model to predict the risk of developing POI for patients undergoing colorectal surgery. After the data were cleaned and assessed for outliers, ML models were applied to the data. Outliers were treated using 1.5 × inter-quantile range (IQR) to identify the 25th to 75th percentile for the continuous numerical dependent variables. Stratified random sampling was used to split the data into training, validation, and testing sets. The dataset was split into 60% training, 20% validation, and 20% testing, stratified by POI. The class distributions were balanced using both oversampling and undersampling techniques. SMOTE was the oversampling technique used to balance the underrepresented class (POI) [8]. Random undersampler was the undersampling technique used to provide an additional approach to balancing the underrepresented class (POI). Missing values in the dataset were treated with scikit Learn k-nearest neighbors imputation (kNN) [8]. The ‘k’ samples were used to estimate the value of the missing datapoints. The missing values in the dataset were imputed using the mean value from the kNN found in the training and validation sets. Although kNN algorithms require continuous data, converting categorical data to numerical data points also satisfies kNN imputation conditions [8].

### 2.4. Model Training and Validation

To determine the best predictive model for our study, we evaluated a range of eight different machine learning models, including decision tree, logistic regression, ensemble, and boosting techniques. This broad selection ensures that we can be adaptable to different learning tasks and data characteristics, allowing us to find the model that best suits our specific research question and dataset.

We trained and cross-validated eight different ML models to identify associations between POI and other variables. These models were: decision tree classifier, which uses a tree-like structure to classify data; logistic regression, which uses a statistical model to model binary dependent variables; bagging classifier, which uses random subsets of the original dataset to aggregate predictions; random forest classifier, which optimizes predictive accuracy while controlling for overfitting; gradient boosting classifier, which trains each weak learner to correct the mistakes of the previous one; adaptive (Ada) boosting classifier; extreme gradient (XG) boosting classifier; and stacking classifier, which combines multiple models to create a stronger predictive model [9]. By using diverse ML models, we can leverage the strengths of different algorithms. Decision trees offer interpretability, while logistic regression provides interpretable coefficients. Ensemble methods like bagging, random forest, and boosting address overfitting concerns, and stacking further improves performance by combining multiple ML models. This comprehensive approach ensures robust and generalizable results. We used five-K-fold cross-validation to train and validate the ML models. We also performed hyperparameter tuning on each ML model to optimize their performance metrics. We then selected the best-performing model with the highest threshold value of the area under the curve receiver operating characteristic curve (AUC ROC curve) for testing on the holdout (test) dataset. To reduce heterogeneity introduced by single random splitting into training and testing sets, we performed bootstrapping on the training dataset. We used simple bootstrapping with replacement to generate new samples called bootstrap resampling. We performed bootstrap resampling on the training dataset 100 times, an adequate threshold for the dataset size (n = 316) [10].

#### Model Descriptions

**Decision Tree Classifier:** This model leverages a tree-like structure for data classification. By iteratively splitting the data based on specific features, the decision tree arrives at a final prediction for each new data point. This approach offers the advantage of interpretability, allowing us to understand the decision-making process behind the model’s predictions [5,8,9,10].

**Logistic Regression:** This well-established statistical method constructs a mathematical model to predict the probability of a binary outcome (POI or no POI in this case). Logistic regression provides interpretable coefficients, revealing the relative influence of each variable on the model’s prediction [5,8,9,10,11].

**Ensemble Methods:**

– **Bagging Classifier:** This technique addresses overfitting concerns by creating multiple, independent decision trees trained on random subsets of the original data. The final prediction is then derived by aggregating the individual predictions from each tree [5,8,9,10].
– **Random Forest Classifier:** Building upon bagging, random forests introduce an additional layer of randomization by randomly selecting a subset of features at each node of the decision trees. This further strengthens the model’s robustness and reduces overfitting [5,8,9,10].
– **Gradient Boosting Classifier:** This sequential ensemble method sequentially trains weak learners (e.g., decision trees) to progressively improve upon the shortcomings of the previous learner. This iterative refinement leads to a more robust final model [5,8,9,10].
– **AdaBoost Classifier (Adaptive Boosting):** This technique builds upon the core idea of boosting by strategically adjusting the weights assigned to training instances during each iteration. Instances that were previously misclassified by the model receive higher weights, forcing the subsequent learners to focus on these challenging cases [5,8,9,10].
– **XGBoost Classifier (Extreme Gradient Boosting):** This advanced boosting algorithm incorporates optimization techniques like regularization to enhance model performance and prevent overfitting. XGBoost is known for its efficiency and scalability when handling large datasets [5,8,9,10].
– **Stacking Classifier:** This meta-learning approach combines the predictions from multiple base learners (e.g., decision trees, logistic regression) to create a final, potentially more accurate prediction. Stacking leverages the strengths of various models to yield a more robust ensemble [5,8,9,10].

### 2.5. Model Performance

The ML model predictions were assessed by confusion matrices. The ML model performance metrics assess sensitivity, specificity, and positive predictive values (PPV). The AUC ROC curve plots true positive rates (sensitivity) against false positive rates (1− specificity). The AUC ROC threshold 0.50 is the result of random predictions and 1.0 as a perfect discriminator [11]. The PPV (precision) is a classifier’s ability to avoid predicting a sample as positive when it is in fact negative. Sensitivity is predicting the sample cases that in fact have the target outcome. Specificity is predicting the sample cases that in fact do not have the target outcome [11]. By incorporating diverse types of ML models, hyperparameter tuning, cross-validation, and AUC ROC curve-based selections ensures a rigorous data-driven approach to identifying the optimal ML model for predicting POI in this study. Statistical analyses were performed in Python 3.9.12, jupyter Notebook 6.4.8, and IBM SPSS Statistics version 26.12 [12].

## 3. Results

### 3.1. Baseline Characteristics

In total, the study found that 6.33% (n = 20) of patients experienced a POI (Table 2). The gender ratio of 2:1 suggests there is a higher proportion of males who experience POI after colorectal surgery, which is consistent with prior studies. The mean age of 62 years indicates POI is more common in older adults; thus, the risk of developing POI increases with age. The mean BMI in the POI group was 30.5 kg/m^2^, within the obese category. 

**Figure 1:**
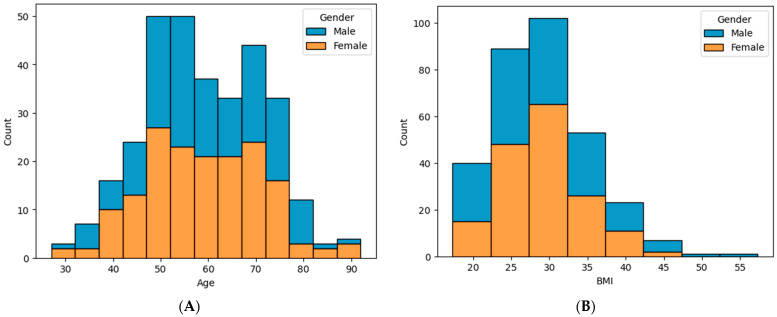
Demographics—Age (**A**) and BMI (**B**) by Gender. Blue: Male, Orange: Female.

Patient demographics such as age, gender, and body mass index (BMI) were extracted from the EHR (Figure 1).

The mean cost of care for patients with POI increased by a factor of 1.77 ± 0.34 compared to patients not experiencing POI. The increased cost of care in the POI patients is partly explained by the increased length of stay from 3.74 (range = 1.0–20.0) days in non-POI patients to 11.64 (range = 6.0–25.0) days in the POI patients (Table 2).

**Figure 2:**
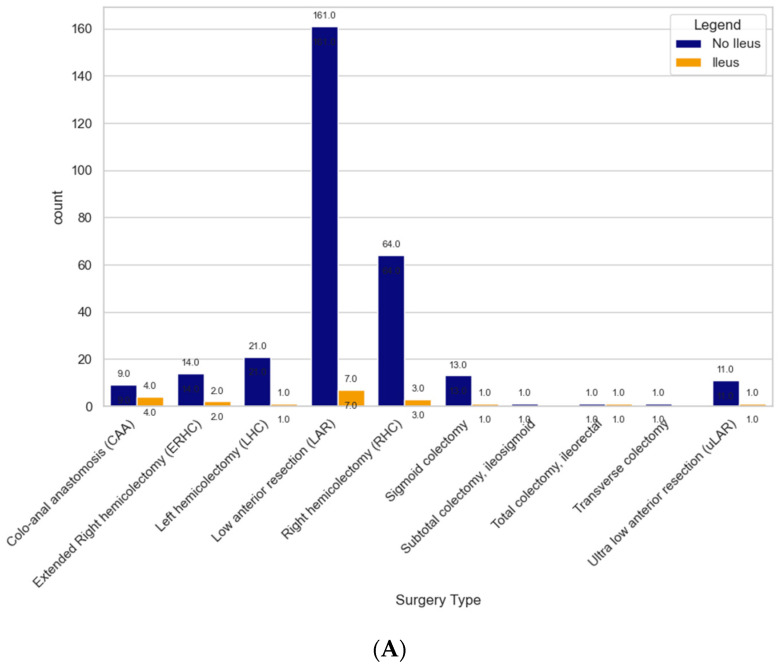

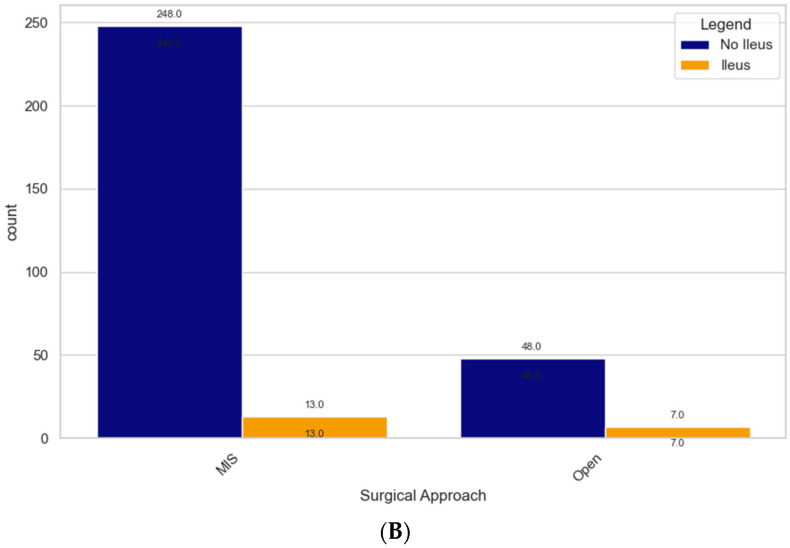
Postoperative Ileus by Surgery Type (**A**) and Surgical Approach (**B**).

For patients undergoing total proctectomy with a coloanal anastomosis (CAA), 44.4% of the sample developed a POI (n = 4) compared to low anterior resection (LAR), after which 0.43% of patients experienced a POI (*p*-value = 0.771) (see Table 2, Figure 2). Based on the surgical approach, patients undergoing minimally invasive surgery (MIS) had a 5.24% rate of POI compared to 14.6% of patients undergoing open surgery (*p*-value = 0.05) (See Table 2, Figure 2).

### 3.2. Comorbidities of Importance

**Figure 3:**
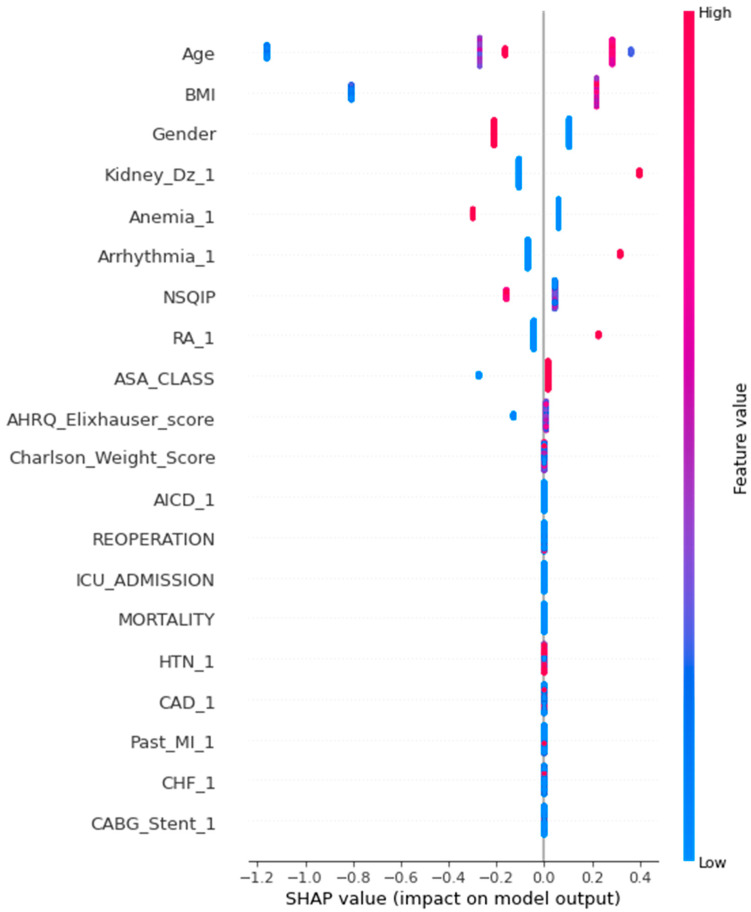
SHAP Summary Plot for Co-Morbidity Contribution to POI.

Overall, the most common comorbidities identified in the sample (n = 316) were electrolyte disturbances 97.5% (n = 308), hypertension 56.3% (n = 178), dyslipidemia 37.3% (n = 118), anemia 24.4% (n = 77), cerebral vascular accident (CVA) 20.6% (n = 65), diabetes 19.9% (n = 63), and coronary artery disease 19.6% (n = 62) (Table 3). The items of importance with the highest feature values contributing to the ML models for POI include age, BMI, gender, kidney disease, anemia, arrhythmia, and rheumatoid arthritis (Figure 3).

### 3.3. ML Model Performance

**Figure 4:**
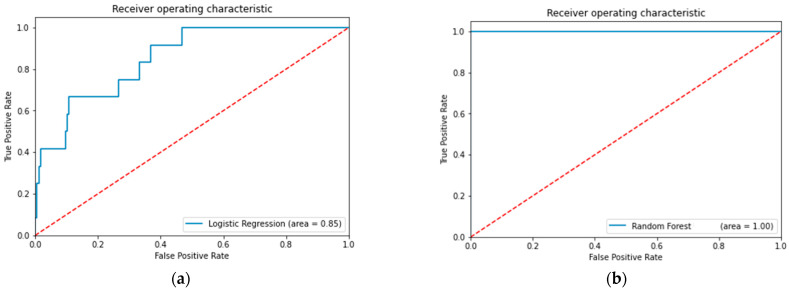

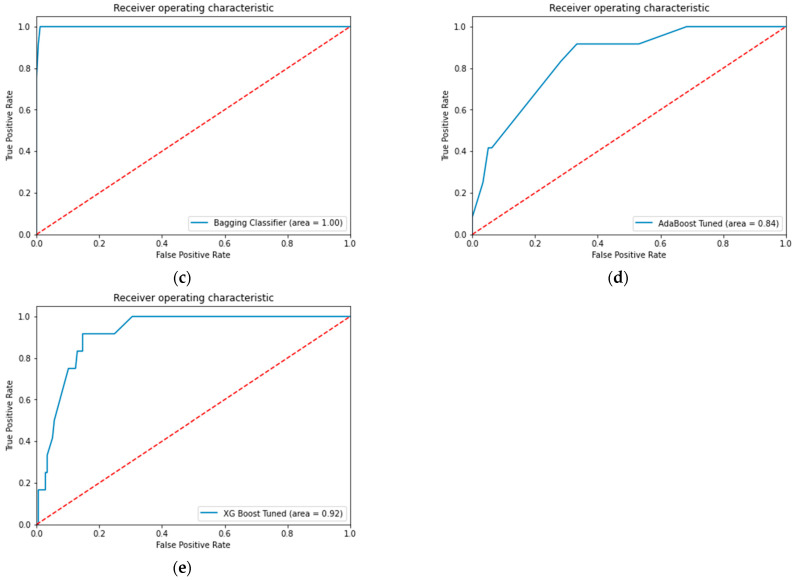
Receiver Operating Characteristics for ML Models Predicting POI. Area under the receiver–operator curve for each machine learning (ML) model was calculated. (**a**) logistic regression model. (**b**) Random forest model. (**c**) Bagging classifier Model. (**d**) AdaBoost (tuned) model. (**e**) XGBoost (tuned) model. ROC curves for grid and random search tuning for both AdaBoost and XGboost classifier models are similar and so not represented in the figure. ROC curves for stacking classifier were overfitted (as were bagging classifier and random forest models—included as an example) and not represented in the figure. The diagonal red line divides the ROC space. Points above the diagonal represent good classification results (better than random); points below the line represent bad results (worse than random).Any model which is overfitted would not be useful for accurate predictions or conclusions, as it would not discriminate from the training data and defeats the very purpose of machine learning. Thus, based on accuracy, only the highest functioning models were included due to overfitting. For example, panel (**b**,**c**) are overfitting [5,9,11,12].

The ability of ML models to predict POI is presented in Figure 4. The ML models incorporated 29 patient comorbidities, four comorbidity risk indices (ASA Physical Status, NISQP, CCI, and ECI), surgery type, and surgical approach in predicting POI. The ML models exhibiting the greatest accuracy were AdaBoost tuned with grid search (94.2%), AdaBoost tuned with random search (94.2%), XG Boost tuned with grid search (85.2%), and XG Boost tuned with random search (85.2%) (Table 4). 

Three of the eight ML models showed good predictive ability (AUC ROC values ≥ 0.84) (See Figure 4). After hyperparameter tuning, random forest and bagging classifier continued to demonstrate oversampling (AUC ROC values = 1.0). Logistic regression, AdaBoost, and XG Boost were strongly predictive for POI (AUC ROC values ≥ 0.84). The hyperparameter-tuned XG Boost models had the strongest predictive ability (AUC ROC values = 0.92), PPV = 0.28, specificity = 0.85, and sensitivity = 0.83 compared to the other models (See Table 4). These findings were consistent with other studies utilizing ML models, such as random forest, AdaBoost, XG Boost, support vector machines, and neural networks for risk stratification predictions on POI [13,14,15]. In this study, the random forest and bagging classifier ML models may have benefited from lasso regularization to further reduce oversampling, which was performed in other studies [13]. Consistent with other large-scale studies, XG Boost with five-K-fold iterations was the predominant ML model providing the best results for predicting POI [13,14,15].

## 4. Discussion

In this study examining POI after colorectal surgery, a 6.33% prevalence was observed, with a male predominance and greater incidence in older adults. POI significantly increased healthcare costs and length of stay. Electrolyte disturbances, hypertension, and dyslipidemia were prevalent comorbidities, while advanced age, high BMI, and male gender emerged as crucial risk factors. CAA and open surgery have an increased risk of developing POI. Several ML models, notably XG Boost with hyperparameter tuning, achieved high accuracy in predicting POI (AUC ROC = 0.92), aligning with existing research on ML-based risk stratification for this complication. The definition of POI remains highly variable throughout the literature [6,16,17,18,19]. For this study, POI was defined as a postoperative event defined by a disturbed gastrointestinal transit time resulting in a range of possible symptoms, including the inability to tolerate oral intake, nausea, vomiting, flatus, and stool abatement [20]. ML models demonstrate good predictive ability with age, BMI, gender, and four comorbidities of importance, namely kidney disease, anemia, arrhythmia, rheumatoid arthritis, and NSQIP risk scores. These conditions lead to electrolyte imbalances, reduced oxygen delivery, and chronic gut inflammation, thus contributing to POI. Therefore, patients with these conditions should be considered at high risk for POI and closely monitored for return of gastrointestinal function [6]. These findings underline the burden of POI, its association with specific comorbidities, type of surgery, surgical approach, and the promising role of ML in its early identification and preventive measures. The pathophysiology of POI is complicated and is associated with patient factors, anesthesia factors (e.g., opioid administration), surgical stress, bowel manipulation, electrolyte disturbances, prolonged surgical times, intravenous fluid administration, and anastomotic leak [20].

In this study, we identified pre-existing electrolyte imbalances or certain medical conditions as underlying vulnerabilities, amplifying the impact of POI on electrolyte homeostasis. These imbalances can manifest as worsening POI symptoms and potentially lead to complications like arrhythmias, muscle weakness, and seizures. These disruptions arise from a multifactorial interplay of factors. Fluid shifts, a hallmark of POI, can concentrate electrolytes in the remaining blood volume, leading to hyponatremia or hyperkalemia. Nutritional deficiencies due to limited oral intake or impaired absorption exacerbate the issue. Therefore, meticulous monitoring of electrolyte levels and timely adjustments to fluids, electrolytes, and medications are crucial for optimal POI management and improved patient outcomes.

Oncologic patients with colorectal cancer undergo a rigorous preoperative evaluation to ascertain the safe delivery of anesthesia and surgical intervention. Enhancing perioperative optimization by identifying risk factors, modifiable and non-modifiable, that may contribute to reducing postoperative morbid outcomes (i.e., POI) offers an opportunity to enhance the quality of care and reduce healthcare costs. Several risk score calculators exist to evaluate perioperative safety and potential postoperative complications. The challenge with existing risk calculators is the inconsistency between the different risk calculators. ASA physical status, a six-level classification system to evaluate comorbidities about potential perioperative risks, measures a patient’s overall health status and risk of complications during surgery [1,21]. The NSQIP score is a risk-adjusted scoring system used to measure the probability of postoperative complications for surgical patients [2,22,23]. The CCI measures a patient’s overall health status and risk of death after surgery. The modified CCI uses preoperative comorbidity data to predict mortality risk at 1 and 10 years [21,22]. The ECI measures a patient’s overall health status and risk of complications. The ECI utilizes preoperative comorbidity data to predict 30-day mortality and readmission risk [24,25].

This study demonstrates that ML may offer new approaches to identifying specific surgery types, surgical approaches, and comorbidities as predictors of POI or other postoperative complications potentially supplementing existing risk index scales in predicting potential comorbidities and overall risk of developing POI after colorectal surgery [13,14]. The hyperparameter tuned XG Boost model provided the best prediction of POI with the highest discrimination on AUC-ROC, PPV, sensitivity, and specificity compared to the other tuned ML models. Since each ML model incorporates a different statistical approach, which can lead to variations in features of importance, a range of ML models should be evaluated when developing risk models for POI. The benefit of ML models over standard regression techniques is the iterative learning ability during model training and when new information is presented to the model [13,14,15]. This can be in the form of a new cohort of patients, or in response to changes in treatment or prevention strategies for the outcome of interest. For example, a new medication correcting anemia or arrhythmias leading to a risk reduction for POI may cause the ML models to adapt in generating new rankings for comorbidity features of importance. 

The importance of preoperative risk stratification and patient optimization is important for predicting and managing postoperative outcomes, like POI [17,18,19]. However, the literature varies on the predictive ability for some risk index models. All risk index models reviewed in the literature suggest age as one of the most important predictors of overall surgical outcomes and complications [13,14,15]. Specific to POI, the literature suggests advanced age and gender are predictors for POI, although in this study the age of patients with POI ranged between 40 to 74 years (mean = 62 years) [26,27,28]. Our findings are consistent with the literature, in that males are more likely to develop POI after colorectal surgery, accounting for 70% (n = 14) of the POI cases. Another less reported comorbidity that we also identified is the effect of BMI on the risk for developing a POI. A prior study identified a BMI ≥ 26 kg/m^2^ as a strong predictor for developing POI, consistent with a median BMI of 30 kg/m^2^ in our POI cohort [19]. Overall, the findings suggest POI is more common in males, older adults, and obese individuals.

The study has several limitations compared to other large-scale studies. The sample size (n = 316) in this study was relatively small, and the incidence of POI was drawn from an administrative billing data set based on ICD-10 codes, which risks underreporting, but this was mitigated to a limited extent by standardized discharge summaries that document each postoperative complication during the hospital stay. A larger sample size may have identified additional features of importance. Accurate incidence of POI or any other complication is best captured through standardized definitions and collected prospectively. However, other studies validate the comorbidities identified by the ML models in this study [13,14,15,17,18,19,27,28]. While this study offers valuable insights into the potential application of ML and natural language processing (NLP) in identifying POI, several limitations underscore the need for caution in interpreting the findings. The retrospective nature of the study, coupled with the small sample size and reliance on EHR, warrants careful consideration of the preliminary nature of the data. Moreover, the low reported incidence of POI raises questions about the methodology used for diagnosis and documentation, highlighting the importance of optimizing EHR and employing advanced techniques like NLP to enhance accuracy and timeliness. The distribution of surgery types and surgical approaches exhibited imbalance within each category, limiting the inference of the risk associated with developing POI. Since the outcome of interest was imbalanced, SMOTE and random undersampler were used to balance the dataset and mitigate the risk of the ML models focusing on the dominant independent and dependent variables. Data quality has a significant influence on ML algorithms. Thus, a concerted effort on exploratory data analysis (EDA) and data preprocessing to ensure data quality was paramount. This study utilized kNN for missing values, which could impact the ML model performance. Although the dataset was retrieved from 2 years of colorectal surgery cases for primary colorectal cancer performed at a high-volume institution, as in all retrospective studies, selection bias could be a factor in the data collection process. ML models accurately predict the risk of POI, but causal relationships between variables and outcomes cannot be interpreted entirely using algorithms alone. The relevance and context of the ML models in clinical practice must be considered for generalizability and broader adoption. Another limitation is the potential anesthesia and intraoperative factors contributing to the incidence of POI. The current analysis focused primarily on patient-related factors associated with POI. While this provides valuable insights, future studies could benefit from incorporating granular data on anesthesia and intraoperative variables, such as anesthetic type, fluid management, and surgical duration, to explore their potential interactions with identified risk factors and identify new modalities for prevention.

A major strength of this study is the utilization of a separate validation set withheld from training and testing [19,29]. This safeguard prevented ML model overfitting on the training data, ensuring a more accurate evaluation of its generalizability on new, unseen data. Consequently, the reported model performance is less prone to bias and more likely to reflect its effectiveness in real-world settings, enhancing confidence in the study’s conclusions.

## 5. Conclusions

This study focused on supervised ML learning models, which achieved similar results to other large-scale studies in identifying comorbidities that may lead to POI. Compared to logistic regression models and similar models, XG boost ML models combine multiple decision tree models, allowing the model to capture complex relationships that linear models can overlook. Specific to complex relationships, XG boost maintains superior accuracy compared to logistic regression models. Additionally, XG boost protects against overfitting by built-in regularization techniques that offer robustness against noise in the data [4,10]. The efficiency and accuracy of ML models provide a new frontier in early detection and intervention for postoperative outcome optimization, real-time predictive analytics, and new research endeavors. Additional research is required to validate and assess the replicability of the results from this study. The ability to identify modifiable comorbidities that may impact perioperative outcomes is promising [30,31]. ML are new tools to help augment clinical decision-making, monitor quality outcomes against benchmarks, and impact perioperative oncological care.

This study lays a foundation for future research aimed at leveraging artificial intelligence to predict and prevent POI. By acknowledging the need for large-scale studies and incorporating intraoperative data, future investigations can build upon this proof of concept to develop more sophisticated ML models. Furthermore, while age and obesity emerged as significant risk factors in this study, the findings underscore the importance of validating results across diverse populations and settings, ensuring robustness and reproducibility in perioperative research. Overall, while acknowledging its limitations, this study paves the way for a strategic expansion of artificial intelligence applications in perioperative care, with the goal of improving patient outcomes and healthcare delivery.

## Figures and Tables

**Table 1:** Flow Diagram for Machine Learning for Imbalanced Datasets.

Data Science Stage	Sub-Stage	Description	Tools/Metrics
Data Acquisition	Source Import	Load data from CSV files, databases, etc.	File paths, data size
	Cleaning and Preprocessing	Check for missing values, inconsistencies, duplicates. Format data types.	Imputation methods, error checking tools
Exploratory Data Analysis (EDA)	Feature Distribution	Analyze data distribution for each feature using histograms, boxplots.	Visualizations, skewness measures
	Feature Relationships	Identify relationships between features and target variable using scatter plots, correlation matrices.	Correlation coefficients, feature importance scores
	Outlier and Bias Detection	Check for outliers and potential biases using boxplots, statistical tests.	Outlier detection algorithms, bias analysis tools
Imbalanced Data Handling	Class Imbalance Assessment	Calculate class imbalance ratio, visualize class distribution using pie charts.	Class imbalance ratio, visualization tools
	Mitigation Strategy Decision	Choose appropriate strategy: SMOTE, undersampling, oversampling, none.	Imbalance severity, data type, problem type
Data Oversampling (Optional)	SMOTE Application	Apply SMOTE or other oversampling techniques to increase minority class.	SMOTE algorithms, minority class size increase
	Oversampling Control	Ensure oversampling does not introduce overfitting or class overlap.	Cross-validation, visualization
Data Undersampling (Optional)	Undersampling Techniques	Apply undersampling techniques to reduce majority class.	Random undersampling, stratified undersampling
	Undersampling Control	Ensure undersampling does not introduce bias or loss of information.	Class balance metrics, cross-validation
Model Selection and Training	Feature Engineering (Optional)	Create new features based on existing ones (ratios, transformations).	Feature engineering algorithms, interpretability measures
	Model Selection	Choose suitable ML algorithms based on data type, problem type, and interpretability needs.	Logistic Regression, Random Forest, Decision Trees, Gradient Boosting, Extreme Gradient Boosting
	Model Training and Regularization	Split data into training, validation, and test sets. Train models with cross-validation and regularization (L1, L2).	Train/validation/test ratios, regularization parameters
Model Evaluation and Testing	Model Validation	Evaluate model performance on validation set using accuracy, precision, recall, F1-score, AUC-ROC (for imbalanced data).	Validation set metrics, model comparison tools
	Best Model Selection	Compare performance across models and select the best one.	Validation metrics comparison, statistical tests
	Model Testing	Evaluate final model on unseen test set to assess real-world performance.	Test set metrics, model generalization analysis
	Error Analysis	Analyze model errors and identify potential limitations.	Error analysis tools, visualization
Production	Interpretation and Deployment	Interpret model results and explain predictions. Deploy model and monitor performance.	Explainable AI tools, model monitoring systems

**Table 2:** Summary of Variables of Importance.

Variable of Importance	No Ileus (n = 296)	SD/Range	Ileus (n = 20)	SD/Range	Chi-Square	*p*-Value
Gender					2.603	0.107
Male	153		14 (70%)			
Female	143		6 (30%)			
Age (mean/SD)	58	+/−12.33	62	+/−10.05		0.055
BMI (median/range)	21.8	17.31–56.10	30.5	20.94–41.53		0.00
NISQP (median/range)	33.6	13.01–46.12	56.2	45.1–78.4		0.00
Length of Stay (Days) (median/range)	3.74	1–20	11.64	6–25		0.00
Cost of Care (Ratio)	1.0	+/−0.36	1.77	+/−0.34		
Co-Morbidity						
Kidney Disease	50		5 (25%)		0.629	0.428
Anemia	77		5 (25%)		0.033	0.855
Arrhythmia	41		4 (20%)		0.458	0.498
Rheumatoid Arthritis	32		4 (20%)		1.613	0.204
Surgical Approach						
Coloanal Anastomosis	9		4 (20%)		0.703	0.402
Extended Right Hemicolectomy	14		2 (10%)		0.259	0.611
Left Hemicolectomy	21		1 (5%)		0.091	0.763
Low Anterior Resection	161		3 (15%)		0.085	0.771
Right Hemicolectomy	64		3 (15%)		0.091	0.763
Sigmoid Colectomy	13		1 (5%)		0.154	0.695
Subtotal Colectomy (Ileosigmoid)	1		0		0.000	0.996
Total Colectomy, Ileorectal	1		1 (5%)		1.047	0.306
Transverse Colectomy	1		0		0.000	0.996
Ultra Low Anterior Resection	11		1 (5%)		0.167	0.683
Surgery Type					3.848	0.050
Minimally Invasive Surgery (MIS)	248 (95%)		13 (5%)			
Open Approach	48 (87.3%)		7 (12.7%)			

**Table 3:** Co-Morbidities for Colorectal Cancer Sample.

Co-Morbidity	Sample Size	Frequency	% of Sample
HTN	316	178	56.3%
CAD	316	62	19.6%
Past MI	316	17	5.4%
CHF	316	25	7.9%
CABG Stent	316	20	6.3%
Arrhythmia	316	41	13.0%
AICD	316	1	0.3%
Pacemaker	316	51	16.1%
Valvular	316	18	5.7%
PVD	316	7	2.2%
Anemia	316	77	24.4%
Diabetes	316	63	19.9%
Hypothyroidism	316	56	17.7%
Electrolyte Disturbance	316	308	97.5%
Asthma	316	60	19.0%
COPD	316	30	9.5%
OSA	316	48	15.2%
CVA	316	65	20.6%
TIA	316	6	1.9%
Seizures	316	7	2.2%
Neuromuscular Disease	316	0	0.0%
Hepatitis	316	26	8.2%
Cirrhosis	316	13	4.1%
AIDS_HIV	316	3	0.9%
Dyslipidemia	316	118	37.3%
Kidney Disease	316	50	15.8%
RA	316	32	10.1%
Depression	316	39	12.3%
Dementia	316	39	12.3%

HTN = hypertension, CAD = coronary artery disease, MI = myocardial infarction, CHF = congestive heart failure, CABG = coronary artery bypass graft, AICD = automatic implantable cardioverter defibrillator, PVD = peripheral vascular disease, COPD = chronic obstructive pulmonary disorder, OSA = obstructive sleep apnea, CVA = cerebral vascular accident, TIA = transient ischemic attack, AIDS_HIV = acquired immunodeficiency syndrome human immunodeficiency virus, RA = rheumatoid arthritis.

**Table 4:** ML Model Performance Comparison.

	AdaBoost Tuned with Grid Search	AdaBoost Tuned with Random Search	XGboost Tuned with Grid Search	XGboost Tuned with Random Search
Accuracy	0.942	0.942	0.852	0.852
Recall	0.083	0.083	0.833	0.833
Precision	1.000	1.000	0.278	0.278
F1	0.154	0.154	0.417	0.417

Accuracy = (True Positive + True Negative)/((True Positive + True Negative + False Positive + False Negative); Recall = True Positive/(True Positive + False Negative); Precision = True Positive/(True Positive + False Positive); F1 = 2 × ((Precision × Recall)/(Precision + Recall)). F1 is the harmonic mean of the precision and recall of any given classification model, which is an indication of model reliability (but this is only one element). For a comparison of the performance of different models, all four characteristics (accuracy, recall, precision, and F1) must be observed. Due to overfitting only the highest functioning models based on accuracy were included [5,9,11,12].

## Data Availability

Study data will not be shared with any individuals or entities that are not involved in the study without IRB approval. No identifying information will be shared with outside collaborating sites or outside collaborating research staff without prior IRB approval, and once a grant/contract and/or a data use or material transfer agreement has been implemented. If approval is obtained, sharing of data would be done after approval of the PI and only by secure mechanisms, as approved by MD Anderson Information Security.

## Abbreviations

Ada boosting classifier	adaptive boosting classifier
ASA physical status	American Society of Anesthesiologists Physical Status
AUC ROC curve	area under the curve receiver operating characteristic curve
BMI	body mass index
CCI	Charlson Comorbidity Index
CVA	cerebral vascular accident
ECI	Elixhauser Comorbidity Index
EHR	electronic health record
EDA	exploratory data analysis
XG boosting classifier	extreme gradient boosting classifier
IQR	inter-quantile range
kNN	k-nearest neighbors imputation
LOS	length of stay
ML	machine learning
NSQIP	National Surgical Quality Improvement Program index
POEM	perioperative evaluation and management
POI	postoperative ileus
PPV	positive predictive values
SMOTE	synthetic minority oversampling technique
UHC	University Health System Consortium
